# Curcumin Ameliorates White Matter Injury after Ischemic Stroke by Inhibiting Microglia/Macrophage Pyroptosis through NF-*κ*B Suppression and NLRP3 Inflammasome Inhibition

**DOI:** 10.1155/2021/1552127

**Published:** 2021-09-30

**Authors:** Yuanyuan Ran, Wei Su, Fuhai Gao, Zitong Ding, Shuiqing Yang, Lin Ye, Xuechai Chen, Guiqin Tian, Jianing Xi, Zongjian Liu

**Affiliations:** ^1^Department of Rehabilitation, Beijing Rehabilitation Hospital, Capital Medical University, Xixiazhuang, Badachu Road, Shijingshan District, Beijing 100144, China; ^2^Department of Neurosurgery, Beijing Tsinghua Chang Gung Hospital, School of Clinical Medicine, Tsinghua University, Beijing 102218, China; ^3^School of Materials Science and Engineering, Beijing Institute of Technology, No. 5 Zhongguancun South Road, Haidian District, Beijing 100081, China; ^4^College of Life Science and Bioengineering, Beijing University of Technology, 100 Ping Le Yuan, Chaoyang District, Beijing 100124, China

## Abstract

NLRP3 inflammasome-mediated pyroptosis is a proinflammatory programmed cell death pathway, which plays a vital role in functional outcomes after stroke. We previously described the beneficial effects of curcumin against stroke-induced neuronal damage through modulating microglial polarization. However, the impact of curcumin on microglial pyroptosis remains unknown. Here, stroke was modeled in mice by middle cerebral artery occlusion (MCAO) for 60 minutes and treated with curcumin (150 mg/kg) intraperitoneally immediately after reperfusion, followed by daily administrations for 7 days. Curcumin ameliorated white matter (WM) lesions and brain tissue loss 21 days poststroke and improved sensorimotor function 3, 10, and 21 days after stroke. Furthermore, curcumin significantly reduced the number of gasdermin D^+^ (GSDMD^+^) Iba1^+^ and caspase-1^+^Iba1^+^ microglia/macrophage 21 days after stroke. *In vitro*, lipopolysaccharide (LPS) with ATP treatment was used to induce pyroptosis in primary microglia. Western blot revealed a decrease in pyroptosis-related proteins, e.g., GSDMD-N, cleaved caspase-1, NLRP3, IL-1*β*, and IL-18, following *in vitro* or *in vivo* curcumin treatment. Mechanistically, both *in vivo* and *in vitro* studies confirmed that curcumin inhibited the activation of the NF-*κ*B pathway. NLRP3 knocked down by siRNA transfection markedly increased the inhibitory effects of curcumin on microglial pyroptosis and proinflammatory responses, both *in vitro* and *in vivo*. Furthermore, stereotaxic microinjection of AAV-based NLRP3 shRNA significantly improved sensorimotor function and reduced WM lesion following curcumin treatment in MCAO mice. Our study suggested that curcumin reduced stroke-induced WM damage, improved functional outcomes, and attenuated microglial pyroptosis, at least partially, through suppression of the NF-*κ*B/NLRP3 signaling pathway, further supporting curcumin as a potential therapeutic drug for stroke.

## 1. Introduction

Stroke remains the leading cause of death and long-term disability all over the world with ischemic stroke accounting for approximately 85% of all cases. Recanalization of the occluded blood vessels with thrombolytic/thrombectomy therapies is the preferred treatment for acute ischemic stroke. However, many ischemic stroke patients are ineligible for these treatments due to the narrow therapeutic time window and the risk of intracerebral hemorrhage. Despite extensive efforts over the past few decades devoted to neuroprotective therapies and the rescue of dying neurons, as well as the development of rehabilitative approaches to repair the stroke-damaged brain, no significant progress has been achieved in the treatment and clinical recovery of stroke [1]. White matter injury is one of the major pathophysiological processes involved in ischemic stroke [2]. In stroke patients, white matter injury, characterized as demyelination and loss of axonal integrity, is an important cause of long-term sensorimotor deficits and cognitive impairments [2, 3]. Compelling evidence shows that endogenous or intervention-dependent white matter repairs, including remyelination, axon preservation, and oligodendrogenesis, can reverse structural disruption, rebuild the neural circuitry, and ultimately restore function [4, 5]. Therefore, a new therapeutic intervention targeting white matter restoration might be a promising strategy to improve clinical recovery outcomes after stroke.

White matter damage and repair are greatly influenced by brain inflammation after stroke [6]. Neuroinflammation, characterized by recruitment of peripheral immune cells, glial activation, and release of cytokines and chemokines, initiates within minutes after acute ischemic stroke onset and persists for long after stroke [7]. Brain inflammation provoked by neural cell death poststroke triggers a cascade of events ultimately resulting in secondary injury and aggravating white matter disruption.

Microglia/macrophage-triggered inflammatory responses play an important role in secondary white matter injury and subsequent white matter repair after stroke [8, 9]. Microglia, the resident immune cells in the central nervous system (CNS), are activated in response to microenvironmental changes and undergo morphological and functional alterations [8–10]. For example, activated microglia can drastically alter their phenotype (so-called “microglial polarization”) with the progression of the ischemic stroke [9]. During the acute phase, microglia/macrophage is polarized toward an early anti-inflammatory and protective phenotype, which is followed by a chronic detrimental proinflammatory type at later stages of the disease. Proinflammatory microglia can secrete proinflammatory cytokines and exacerbate oligodendrocyte cell death and demyelination, thereby aggravating white matter injury [11]. In contrast, anti-inflammatory polarized microglia can produce beneficial mediators and promote remyelination, thus facilitating white matter repair [12]. Besides, microglial pyroptosis, a type of proinflammatory programmed cell death, also participates in stroke-induced inflammatory responses [13]. Pyroptosis is dependent on inflammasome activation, driven by the binding of cytoplasmic sensor proteins to pathogen-associated molecular patterns (PAMPs) or damage-associated molecular patterns (DAMPs) [14]. Inflammasome complexes usually consist of NOD-like receptors containing pyrin domains (NLRPs), the adapter apoptosis-associated speck-like protein containing the CARD (ASC), and caspases. Typically, oligomerization of NLRP3 with the ASC and pro-caspase-1 activates the NLRP3 inflammasome, leading to caspase-1 cleavage. Activated caspase-1 subsequently cleaves pro-IL-1*β* and pro-IL-18 into mature IL-1*β* and IL-18, respectively [14]. In addition, activated caspase-1 also cleaves GSDMD into a N-terminal fragment of GSDMD (GSDMD-N). Activated GSDMD translocates to the plasma membrane, binds to the inner membrane lipids, and eventually forms membrane pores, which cause the release of intracellular IL-1*β* and IL-18 and lead to proinflammatory responses [15]. Emerging evidence suggests that NLRP3 inflammasome-mediated microglial pyroptosis plays a crucial role in brain inflammation and functional recovery after stroke [16, 17]. For example, melatonin-treated exosomes effectively improved functional recovery through the suppression of microglial pyroptosis after brain ischemia. Therefore, inhibition of inflammasome-dependent microglial pyroptosis might prove a new strategy for white matter repair after stroke.

Curcumin (1,7-bis[4-hydroxy-3-methoxyphenyl]-1,6-heptadiene-3,5-dione) is the predominant curcuminoid in *Curcuma longa* and has been shown to directly exert protective effects against cerebral ischemia through multiple mechanisms, e.g., inhibiting mitochondrial apoptosis [18] and endoplasmic reticulum stress [19] and stimulating neurogenesis [20]. Our group [21] and others [22] revealed that curcumin treatment has indirect neuroprotective effects through shifting microglia/macrophage polarization from a proinflammatory phenotype to an anti-inflammatory state. However, the effects of curcumin on microglial pyroptosis and white matter integrity after stroke have not been explored.

In this study, we assessed whether poststroke administration of curcumin could protect against white matter damage in an animal model of transient middle cerebral artery occlusion/reperfusion (MCAO/R). In detail, the inhibitory effects of curcumin treatment on microglial inflammasome activation and pyroptosis were examined both *in vivo* and *in vitro*. Mechanistically, the impacts of curcumin on NF-*κ*B pathway activation and downstream effects on NLRP3 inflammasome were investigated.

## 2. Materials and Methods

### 2.1. Animal

Adult male C57BL/6J mice (8-10 weeks, weight 23-25 g) were purchased from the Charles River Laboratory (Beijing, China). Mice were housed in temperature (23 ± 2°C)- and humidity (40-60%)-controlled cages with a 12-hour light-dark cycle, with food and water *ad libitum*. Animal experiments were performed according to the recommendations of the Animal Ethical Committee of Beijing Rehabilitation Hospital, Capital Medical University (approval ID: 2019bkky-08-012; Aug 2, 2019). All efforts were made to minimize animal suffering in accordance with the guidelines of the Institutional Animal Care and Use Committee (IACUC). Mice were randomly assigned into the sham, MCAO+vehicle, and MCAO+curcumin groups (*n* = 8). All mice were used for multiple outcome studies, including infarct volume, neurobehavioral tests, white matter injuries, and protein expression.

### 2.2. Models of Transient Cerebral Ischemia and Stereotaxic Microinjection

A transient middle cerebral artery occlusion/reperfusion (MCAO/R) model was produced by intraluminal occlusion of the right middle cerebral artery (MCA) for 60 minutes [11]. During the operation, mice were anesthetized with 1.5% isoflurane in a 30% O_2_/70% N_2_ mixture. A commercial monofilament (Doccol, 602156PK5Re, USA) was inserted into the origin of the right MCA from the right common carotid artery (CCA) and kept in MCA for 60 minutes. Rectal temperature was maintained at 37 ± 0.5°C throughout the surgery using a temperature-controlled heating pad. A two-dimensional laser speckle imager (Perimed AB, Järfälla, Sweden) was used to evaluate regional cerebral blood flow (rCBF), and animals with rCBF reduction less than 70% of preischemia baseline levels were excluded. The sham mice underwent the same anesthesia and surgery, but without MCA occlusion.

Both AAV-shRNA-NLRP3 and negative control for nonsequence-specific effects were designed and synthesized by GeneChem (Shanghai, China). The nucleotide sequences of AAV-shRNA-NLRP3 are listed in Table 1. C57BL/6J (8 weeks old) mice received one microinjection with either the AAV-shRNA-control or AAV-shRNA-NLRP3 mixture (1 *μ*L of 1 × 10^9^ viral genomes/*μ*L) in the ipsilateral cortex ischemic lesions, as previously reported [23]. The coordinates of the microinjection were the following: 0.3 mm anterior to the bregma, 3 mm lateral, 2 mm deep (AP: 0.3, ML: 3, depth: 2) and 1.9 mm posterior to the bregma, 3 mm lateral, 2 mm deep (AP: -1.9, ML: 3, depth: 2). The injection was completed with a 10 *μ*L microsyringe at the rate of 0.1 *μ*L/min, and the microinjector was placed for 10 minutes after injection and then withdrawn. To evaluate the effects of NLRP3 on pyroptosis in the ischemic stroke mouse model, mice were then subjected to MCAO operation, 2 weeks after AAV microinjection, and divided into the following four groups (*n* = 5): MCAO+shRNA-Con+vehicle, MCAO+shRNA-Con+curcumin, MCAO+shRNA-NLRP3+vehicle, and MCAO+shRNA-NLRP3+curcumin groups.

### 2.3. Drug Preparation and Treatments

A stock solution of curcumin (Sigma-Aldrich, USA) was prepared in 5 M NaOH, pH 7.4 (adjusted with 1 M HCl), and further diluted in 0.9% normal saline for *in vivo* studies [21]. Curcumin (150 mg/kg) or vehicle was administered intraperitoneally immediately after reperfusion and continuously injected for 7 days after ischemic stroke. The same concentration of solvent was used as the vehicle control. For *in vitro* studies, the stock solution of curcumin was diluted in culture medium. Primary microglial cells were treated with curcumin (12.5 *μ*M) or vehicle for 24 hours together with or without LPS (100 ng/mL, Sigma, USA) and then stimulated with ATP (1 mM, Sigma, USA) for another 3 hours. The doses of curcumin were selected based on existing literature [21].

### 2.4. Adhesive Removal Test

The adhesive removal test was used to evaluate the effects of curcumin (*n* = 8 per group) on tactile responses and sensorimotor asymmetries up to 21 days after ischemic stroke, as previously described [21]. In a nutshell, the tactile sensation of the left forelimb is stimulated with an adhesive tape (0.3 × 0.4 cm^2^), and investigators, blinded to the experimental groups, recorded the time to contact and removal of the tape to a maximum of 120 seconds per trial. Preoperative training of each mouse was carried out for three days, and tests were performed presurgery and 3, 10, and 21 days after ischemic stroke.

### 2.5. Infarct Volume Measurements

Three days after MCAO, 2,3,5-triphenyltetrazolium chloride (TTC) staining was performed to evaluate the infarct volume. Briefly, mice were anesthetized with sodium pentobarbital (50 mg/kg, *i.p*.) and humanly sacrificed. Brains were dissected and sliced into six 1 mm thick sections. Sections were then stained with 2% TTC in saline as previously reported [21]. Infarct volume was analyzed using ImageJ software and calculated as the percentage (%) of (the volume of the contralateral area minus the noninfarcted volume of the ipsilateral region)/(the volume of the contralateral area × 2) × 100%.

### 2.6. Detection of Brain Tissue Loss

Microtubule-associated protein 2 (MAP-2) immunostaining was performed to evaluate brain tissue loss. At 21 days after surgery, mice (*n* = 6 per group) were anesthetized with pentobarbital sodium (50 mg/kg, *i.p*.) and transcardially perfused with 0.9% saline. Brains were removed and fixed with 4% paraformaldehyde (PFA) overnight at 4°C and then dehydrated in 20% to 30% sucrose solutions. Serial coronal sections (20 *μ*m) were cut with a freezing microtome (Leica, Germany). The sections were washed in phosphate-buffered saline (PBS) and incubated with 10% normal goat serum (ZSGB-BIO, China) for 60 minutes at room temperature. The sections were then incubated with rabbit anti-MAP-2 antibody (1 : 1000, AB221693, Abcam) overnight at 4°C. After washing with PBST, the samples were further incubated with donkey anti-rabbit IgG-CFL 488 (1 : 100, sc-362261, Santa Cruz) for 2 hours at room temperature. All samples were examined under a laser confocal microscope (Nikon, Japan). The analysis and calculation method of brain tissue loss were consistent with the measurement method of infarct volume.

### 2.7. Tissue Preparation and Luxol Fast Blue (LFB) Staining

Brain slices were stained with Luxol fast blue (LFB) according to the manufacturer's instructions to reveal white matter (WM) injuries. In brief, coronal sections were incubated with the solution of LFB (Solarbio, Beijing, China) at room temperature for 12-20 hours. After washing with water, sections were treated with Luxol differentiation solution (lithium carbonate solution) for 15 seconds, followed by 70% ethyl alcohol for 30 seconds. Sections were washed, dehydrated, and covered for microscopic examination. Images of corpus callosum and striatum were taken with a light microscope (Olympus, Japan).

### 2.8. Immunofluorescence Staining and Analysis

The pyrocytosis of microglia was detected by double immunofluorescence staining. In brief, brain sections were fixed in 4% PFA, washed in PBS, and permeabilized by 0.25% Triton-X100 in PBS. After blocking with 10% normal goat serum (ZSGB-BIO, China), the sections were incubated with a primary antibody mixture containing rabbit anti-Iba-1 antibody (1 : 500, ab178846, Abcam), mouse anti-GSDMD antibody (1 : 50, sc-393581, Santa Cruz)/mouse anti-caspase-1 antibody (1 : 50, sc-392736, Santa Cruz) for 36 hours at 4°C, and then incubated with a cocktail of solution mixture of mouse anti-rabbit IgG-CFL 594 (1 : 100, sc-516250, Santa Cruz) and mouse-IgG*κ* BP-CFL 488 (1 : 50, sc-516176, Santa Cruz) for 4 hours at room temperature. Samples were counterstained with 4,6-diamidino-2-phenylindole (DAPI) (Southern Biotech, USA) and acquired with a laser confocal microscope (Nikon, Japan). Brain sections were processed for automated analysis with ImageJ software, and quantification of positive cells was blinded to experimental groupings. The numbers of positive cells in the cortex (CTX), corpus callosum (CC), and striatum (STR) were calculated per square millimeter from three random microscopic fields (200x magnification) on eight sections (nine images total) (*n* = 8 per group).

### 2.9. Primary Microglia Culture and Cell Model

Primary microglia were isolated from the whole brains of neonatal C57BL/6J mice at P1-P2 and cultured as previously described [24]. After 10 days of initial culture, microglia were shaken off, collected, and incubated in DMEM/F12 medium (Gibco, Life Technologies, USA) with 10% fetal bovine serum (Gibco, Life Technologies, USA) and 1% streptomycin/penicillin (Gibco, Life Technologies, USA). Microglial cells were maintained at 37°C under 5% CO_2_ and treated with vehicle or curcumin (12.5 *μ*M) in the presence or absence of LPS (100 ng/mL, Sigma, USA) and incubated for 24 hours. For LPS+ATP stimulation, ATP (1 mM, Sigma, USA) was added for 3 hours following LPS stimulation. Microglia were collected 27 hours after initial seeding for mRNA and protein analysis. Cell experiments were performed three times independently.

### 2.10. NLRP3 siRNA Transfection

Small interfering RNA (siRNA) targeted at mouse NLRP3 was used to silence NLRP3. The NLRP3 siRNA and nonsense control (NC) were synthesized by Gene Pharma (Suzhou, China). The sequence of siRNA is 5′-CGGCCUUACUUCAAUCUGUTT-3′ (forward) and 5′-ACAGAUUGAAGUAAGG CCGTT-3′ (reverse). The sequence of nonsense control siRNA is 5′-UUCUCCGAACGUGUCACGUTT-3′ (forward) and 5′-ACGUGACACGUUCG GAGAATT-3′ (reverse). Briefly, microglial cells were seeded in 24-well plates and cultured in complete culture medium without streptomycin/penicillin. Cells were transiently transfected with NLRP3 siRNA or negative control using Lipofectamine 3000 (Invitrogen). After transfection for 24 hours, cells were stimulated with curcumin (12.5 *μ*M) or LPS (100 ng/mL) for 24 hours, followed by ATP (1 mM) for another 3 hours.

### 2.11. RNA Extraction and Quantitative Real-Time Polymerase Chain Reaction (qRT-PCR)

Total RNA of primary microglia was extracted with the TRIzol reagent (Invitrogen, Carlsbad, CA, USA) according to the manufacturer's protocol [25]. Briefly, cells were lysed by vortexing in the TRIzol reagent. Chloroform was then added to the cellular lysates for phase separation into an aqueous phase and an organic phase, and isopropanol was used for RNA precipitation. After washing with 75% ethanol, RNA was dissolved with RNase-free water and further quantified using NanoDrop (Thermo Fisher Scientific, USA).

Total RNA was reverse transcribed into cDNA with iScript cDNA synthesis kits (Bio-Rad, Hercules, CA, USA) in a final volume of 20 *μ*L. Quantitative real-time PCR (qRT-PCR) with Sofast EvaGreen Supermix (Bio-Rad) was performed on a 7500 fast real-time PCR platform (Applied Biosystems, Foster City, CA, USA). GAPDH was used as an internal loading control. The expression of target genes was normalized to the GAPDH gene and reported as 2^−*ΔΔ*CT^ [25]. The primer sequences are summarized in Table 2 (Invitrogen).

### 2.12. Western Blot

The brain tissue in the peri-infarct regions and primary microglial cells were used for western blot analysis as previously described [11]. Total protein content of microglia was extracted in RIPA buffer (Beyotime, China) supplemented with the protease/phosphatase inhibitors (Roche). Brain tissues in the peri-infarct regions were homogenized and lysed in ice-cold RIPA buffer solution for protein extraction. The concentration of protein was measured using the BCA protein assay kit (Thermo Fisher) according to the manufacturer's instructions. Each sample extract was then analyzed with 10% or 12% SDS-PAGE gel electrophoresis, blotted onto a PVDF membrane (Bio-Rad), and blocked with fat-free milk (10%, Bio-Rad). The PVDF membranes were then incubated overnight at 4°C with the primary antibodies (Table 3). After washing, membranes were incubated for 1 hour at room temperature with secondary detection horseradish peroxidase-labeled antibodies anti-mouse (1 : 10000, ab6728, Abcam) or anti-rabbit secondary antibody (1 : 10000, ab6721, Abcam). Glyceraldehyde phosphate dehydrogenase (GAPDH) was used as an internal control for quantification. Reactivity was visualized with enhanced chemiluminescence (ECL) kits (Millipore, Germany) and captured by ECL detection systems (Bio-Rad). The relative density value of each band was measured with ImageJ.

### 2.13. Statistical Analysis

Statistical analyses were performed using Prism software version 7.0 (GraphPad Software, San Diego, CA, USA). All data were expressed as the mean ± standard error of mean (SEM). Comparison of two groups was performed using Student's *t*-test. Multiple comparisons were done using the one-way or two-way ANOVA followed by the Bonferroni post hoc test. All tests were considered statistically significant at *p* < 0.05.

## 3. Results

### 3.1. Curcumin Treatment Ameliorates Infarct Volume and White Matter Damage and Promotes Sensorimotor Functional Recovery after MCAO

**Figure 1:**
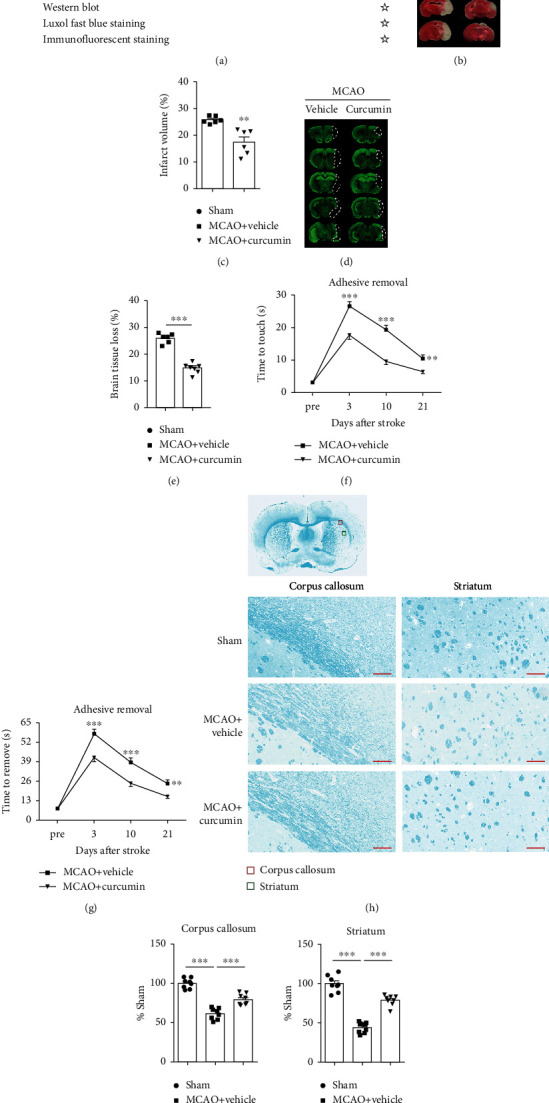
Curcumin treatment significantly reduces white matter injury and improves sensorimotor function after ischemic stroke. Mice were subjected to middle cerebral artery occlusion (MCAO) and then treated with curcumin (150 mg/kg, *i.p*.) immediately after reperfusion and once daily for 7 days after ischemia. (a) Illustration of the *in vivo* experimental timelines. (b) Representative TTC staining and (c) quantification of the infarct volume at 3 days after MCAO. *n* = 6 for each group. ^∗∗^*p* < 0.01. Student's *t*-test. (d) Representative MAP-2 staining and (e) quantification of brain tissue loss at 21 days after MCAO. *n* = 6 for each group. ^∗∗^*p* < 0.01. Student's *t*-test. (f, g) Adhesive removal was used to evaluate long-term sensorimotor deficits at pre-MCAO and 3, 10, and 21 days after surgery. (f) Time to touch adhesive tapes; (g) time to remove adhesive tapes, *n* = 8 for each group. Data are presented as means ± SEM. ^∗∗^*p* < 0.01, ^∗∗∗^*p* < 0.001. Two-way ANOVA followed by Bonferroni post hoc test. (h) Schematic diagram illustrating the anatomical location of images in the ipsilateral perilesion corpus callosum (red) and striatum (green). Representative Luxol fast blue (LFB) staining in the ipsilateral peri-infarct corpus callosum (CC) and striatum areas 21 days after MCAO. Scale bar = 50 *μ*m. *n* = 8 for each group. (i, j) The integrated density of LFB staining was quantified and normalized to the sham group. ^∗∗^*p* < 0.05 vs. vehicle. One-way ANOVA followed by Bonferroni *post hoc* test.

To verify the beneficial effects of curcumin on white matter damage and functional outcome poststroke, infarction was induced in mice through MCAO and then treated with curcumin intraperitoneal injection immediately after reperfusion. Treatment was continued for 7 days after surgery with daily administrations (Figure 1(a)). Neuroprotective effects of curcumin against stroke were evaluated by acute infarct volumes at 3 days and long-term brain tissue loss at 21 days after MCAO. Curcumin posttreatment significantly reduced the infarct volumes compared to the vehicle-treated group 3 days poststroke (Figures 1(b) and 1(c)). Meanwhile, the brain tissue loss in curcumin-treated mice was significantly smaller than that of vehicle-treated mice at 21 days after MCAO (Figures 1(d) and 1(e)). Our previous study revealed that curcumin treatment improved neurological function during the early disease stage in a distal MCAO model [21]. Therefore, we investigated whether curcumin treatment ameliorates long-term sensorimotor deficits after ischemic stroke using the adhesive removal test. Our data show that MCAO prolonged times to touch and removal of the tapes up to 21 days poststroke. Curcumin injection markedly reduced these times during the testing period (Figures 1(f) and 1(g)). These results show that treatment with curcumin remarkably conferred protection from the early stage up to long-term phases after stroke.

We further examined whether curcumin treatment could alleviate white matter damage by LFB staining after stroke. As shown in Figure 1(h), the optical density (OD) value of LFB-stained areas in both corpus callosum (CC) and striatum was dramatically reduced in vehicle-treated stroke mice 21 days after MCAO. Contrastingly, curcumin treatment significantly increased OD values of LFB-stained white matter compared to the vehicle control (Figures 1(i) and 1(j)). LFB staining showed significant suppression of stroke-induced demyelination with curcumin treatment.

### 3.2. Curcumin Treatment Reduces Cerebral Ischemia-Induced Pyroptosis *In Vivo*

**Figure 2:**
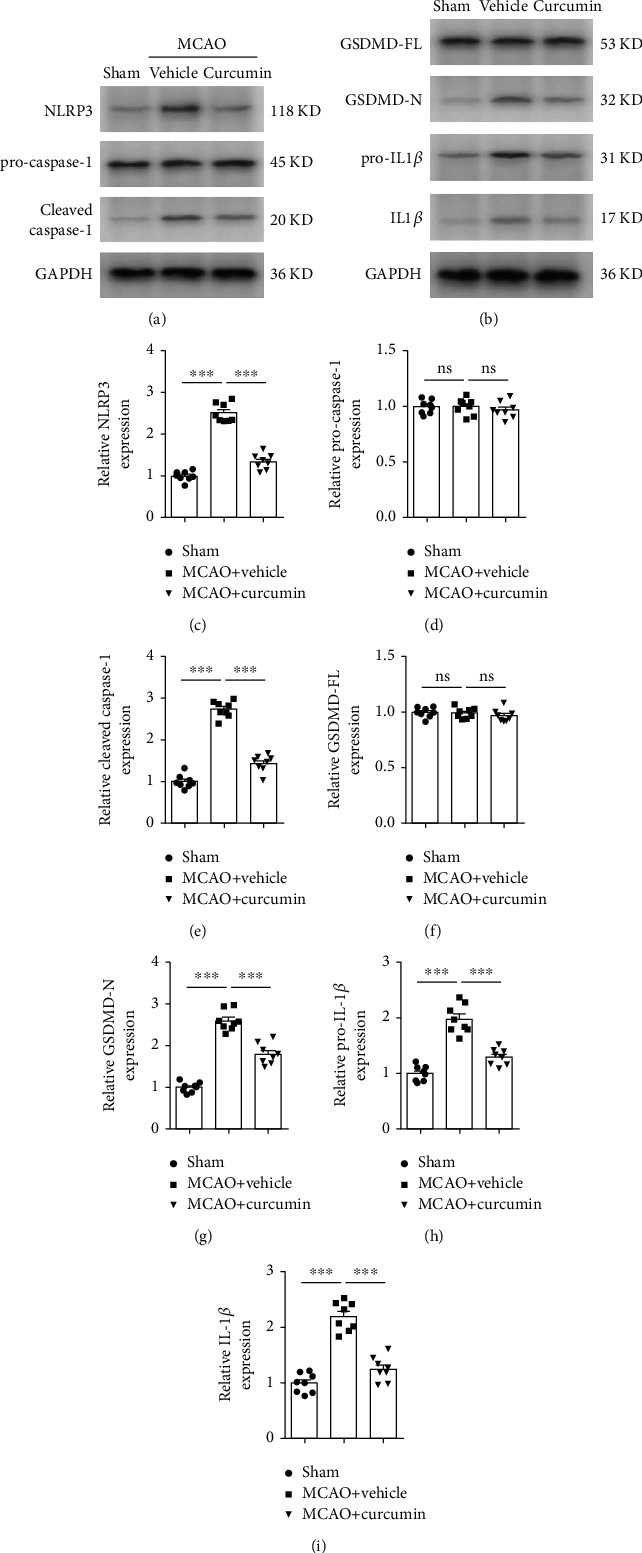
Curcumin treatment reduces stroke-mediated NLRP3 inflammasome activation and pyroptosis in the ipsilateral peri-infarct regions 21 days after cerebral ischemia. (a, b) Representative western blot of NLRP3 and pyroptosis-related proteins in mice. Quantification of western blot data of NLRP3 (c), pro-caspase-1 (d), cleaved caspase-1 (e), GSDMD-FL (f), GSDMD-N (g), pro-IL-1*β* (h), and IL-1*β* (i). All the values are the mean ± SEM. ^∗∗∗^*p* < 0.001. *n* = 8 mice per group, 1 band/mouse. One-way ANOVA followed by Bonferroni post hoc test. GSDMD-FL denotes GSDMD-full length. GSDMD-N indicates GSDMD-N-terminal.

Microglial pyroptosis can promote the release of intracellular IL-1*β* and IL-18, contributing to proinflammatory responses after stroke. The inhibition of microglial pyroptosis was reported to attenuate stroke-damage brain and promote functional outcomes [16, 17]. To determine whether curcumin could reduce ischemia-induced pyroptosis, we analyzed the levels of pyroptosis-related proteins including GSDMD-N, the activated form of GSDMD, as a pyroptotic marker in the peri-infarct regions. As expected, MCAO significantly induced the expression of the cleaved GSDMD (GSDMD-N) 21 days after stroke (Figures 2(b) and 2(g)). Importantly, treatment with curcumin inhibited the expression of GSDMD-N after stroke, indicative of pyroptosis inhibition. No significant changes in the expression of GSDMD-full length (GSDMD-FL) were observed among the three groups (Figures 2(b) and 2(f)). NLRP3 inflammasome is an upstream signal of GSDMD cleavage, and its activation can result in cell pyroptosis [17]. Therefore, we next examine the effects of curcumin on NLRP3 inflammasome activation. Western blot results showed that the expressions of both NLRP3 and cleaved caspase-1 were greatly increased after stroke. However, these were suppressed by curcumin treatment (Figures 2(a), 2(c), and 2(e)). Finally, we measured the expression of pro-IL-1*β* and IL-1*β* in perilesion regions in stroke brains. Stroke also induced noticeable increases in the pro-IL-1*β* and IL-1*β*, which were again dramatically suppressed by curcumin treatment (Figures 2(b), 2(h), and 2(i)). Our data suggest that curcumin suppressed proinflammatory responses following ischemic stroke, at least in part, through pyroptosis inhibition.

### 3.3. Curcumin Suppresses Stroke-Induced NLRP3 Inflammasome Activation and Microglial Pyroptosis *In Vivo* and *In Vitro*

**Figure 3:**
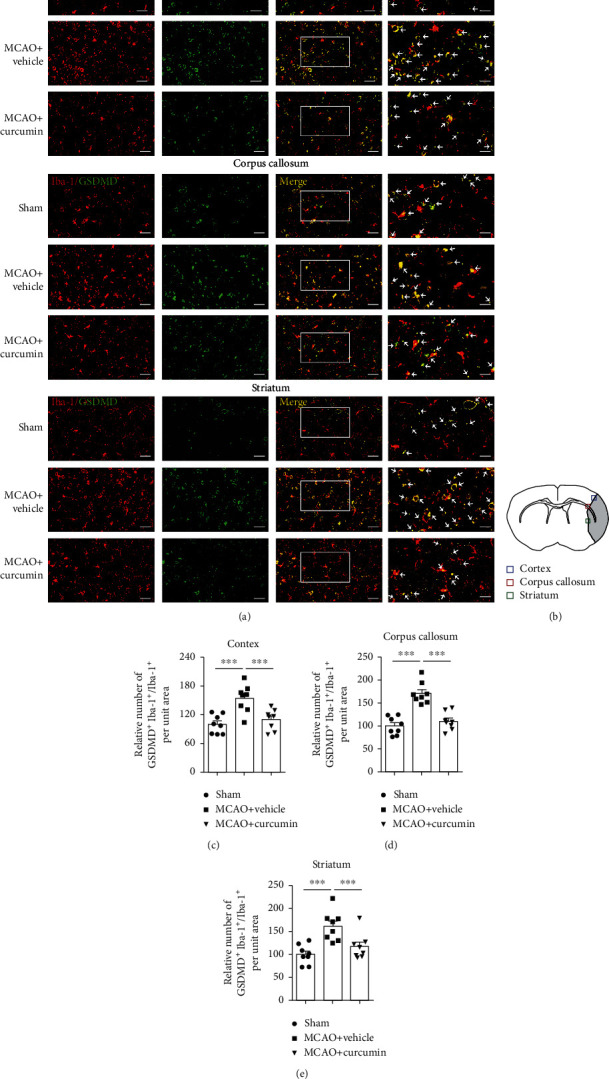
Curcumin treatment reduces GSDMD-positive microglia in the ischemic cortex, corpus callosum, and striatum 21 days after MCAO. (a) Representative coimmunostaining of Iba-1 (red) and GSDMD (green). Scale bar in the figures of 1-3 columns from left = 20 *μ*m, and scale bar in the figure of the 4th column from left = 10 *μ*m. Arrows indicate GSDMD^+^Iba-1^+^ microglia. (b) Schematic diagram illustrating the anatomical location of images in the ipsilateral perilesion cortex (blue), corpus callosum (red), and striatum (green). (c–e) Quantitative analysis of GSDMD-positive microglia. Quantification of the percentage of GSDMD^+^Iba-1^+^ cells among total Iba-1^+^ cells in the perilesion cortex (c), corpus callosum (d), and striatum (e). Values are the mean ± SEM. ^∗∗∗^*p* < 0.001. *n* = 8 mice per group. One-way ANOVA followed by Bonferroni post hoc test.

**Figure 4:**
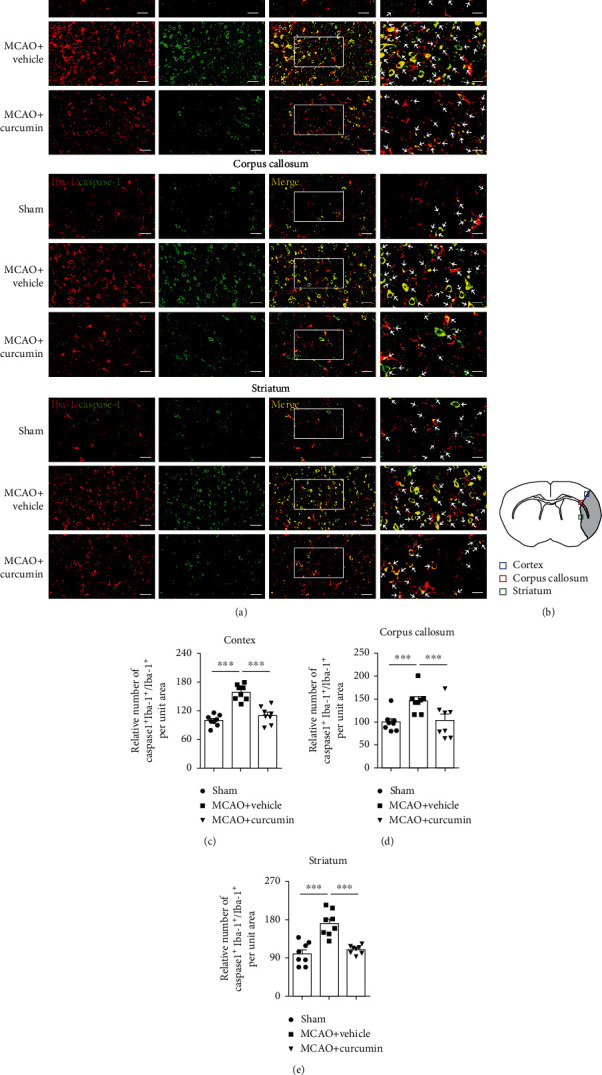
Curcumin treatment attenuates caspase-1 positive microglia in the ischemic cortex, corpus, and striatum at 21 days after MCAO. Representative immunofluorescence costaining for caspase-1 and Iba-1 markers in brain sections obtained from the curcumin- or vehicle-treated groups. (a) Costaining for Iba-1 (red) and caspase-1 (green) in the perilesion cortex, corpus callosum, and striatum. Scale bar in the figures of 1-3 columns from left = 20 *μ*m, and scale bar in the figure of the 4th column from left = 10 *μ*m. Arrows indicate caspase-1^+^Iba-1^+^ microglia. (b) Schematic diagram illustrating the anatomical location of images in the perilesion cortex (blue), corpus callosum (red), and striatum (green). Quantification of the percentage of caspase-1^+^Iba-1^+^ cells among total Iba-1^+^ cells in perilesion cortex (c), corpus callosum (d), and striatum (e). Values are the mean ± SEM. ^∗∗∗^*p* < 0.001. *n* = 8 mice per group. One-way ANOVA followed by Bonferroni *post hoc* test.

Proinflammatory pyroptosis in the ischemic brain is mainly mediated by microglia [26]. To confirm the occurrence of pyroptosis in microglia, we quantified the frequency of GSDMD^+^ or caspase-1^+^ in Iba1^+^ microglia/macrophage by immunofluorescence costaining (Figures 3(a) and 4(a)). Immunofluorescent labeling showed an increase in GSDMD^+^ microglial cells in the ipsilateral cortex, corpus callosum, and striatum regions, 21 days poststroke (Figures 3(c)–3(e)), compared to the sham-treated group. Curcumin treatment markedly suppressed GSDMD immunoreactivity in MCAO mice. In addition, MCAO increased the frequency of caspase-1^+^ cells in Iba1^+^ microglia/macrophage in these three areas, which was significantly inhibited 21 days after stroke and curcumin administration (Figures 4(c)–4(e)). These *in vivo* findings provided solid evidence that curcumin suppressed stroke-induced microglial pyroptosis and caspase-1 activation.

**Figure 5:**
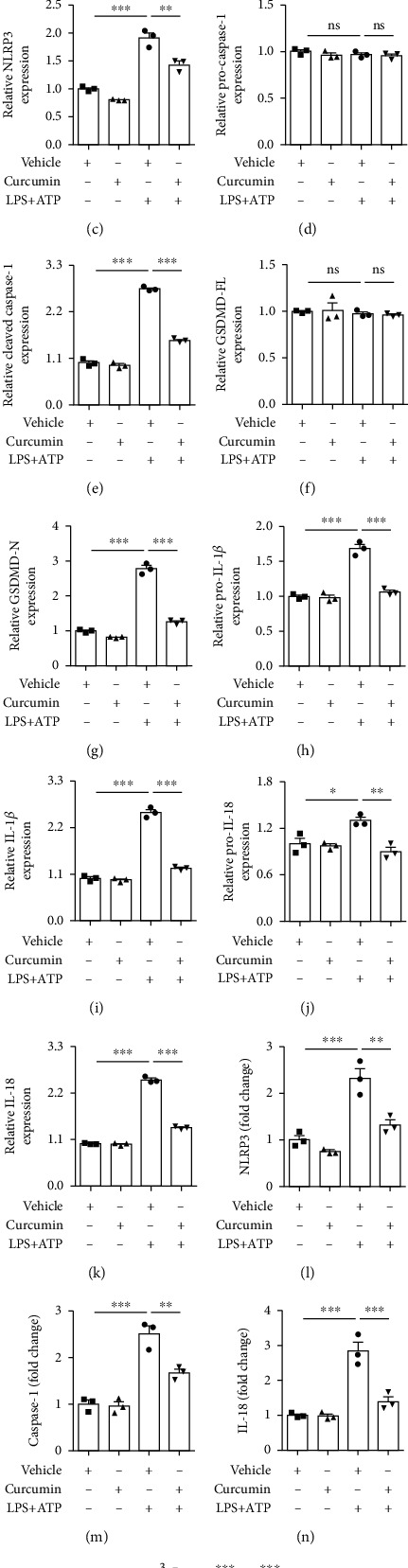
Curcumin inhibits microglial pyroptosis in lipopolysaccharide- (LPS-) and ATP-stimulated primary microglial cells. Primary microglia were extracted from the cortex of newborn C57BL/6J mice. Microglia were treated with vehicle or curcumin (12.5 *μ*M) in the presence or absence of LPS (100 ng/mL) for 24 hours followed by ATP (1 mM) stimulation for further 3 hours. (a, b) Representative western blot of pyroptosis-related proteins. Quantitative analysis of western blot data for NLRP3 (c), pro-caspase-1 (d), cleaved caspase-1 (e), GSDMD-FL (f), GSDMD-N (g), pro-IL-1*β* (h), IL-18 (i), pro-IL-18 (j), and IL-18 (k). All the data are means ± SEM. *n* = 3 per group. Samples were collected from three independent experiments. ^∗^*p* < 0.05, ^∗∗^*p* < 0.01, and ^∗∗∗^*p* < 0.001, one-way ANOVA followed by Bonferroni post hoc test. The mRNA expression of NLRP3 (l), caspase-1 (m), IL-18 (n), and IL-1*β* (o) was examined by real-time PCR. All the data are means ± SEM. *n* = 3 per group. Samples were collected from three independent experiments. ^∗∗^*p* < 0.01 and ^∗∗∗^*p* < 0.001, one-way ANOVA followed by Bonferroni *post hoc* test.

Next, we assessed the impacts of curcumin on primary microglial pyroptosis *in vitro*. Western blot data showed that LPS+ATP stimulation significantly increased the expressions of NLRP3, cleaved caspase-1, and GSDMD-N in primary microglia (Figures 5(a), 5(c), 5(e), and 5(g)). Real-time PCR indicated that the mRNA level of NLRP3 and caspase-1 was greatly elevated in primary microglia following LPS+ATP treatment (Figures 5(l) and 5(m)). These results validated that LPS+ATP induced NLRP3 inflammasome activation and pyroptosis in microglia. In accordance with *in vivo* results, curcumin treatment significantly inhibited the activation of NLRP3 inflammasome and microglial pyroptosis, which were induced by LPS+ATP. Meanwhile, curcumin markedly inhibited the increased protein expression of pro-IL-1*β*, IL-1*β*, pro-IL-18, and IL-18 in microglia following LPS+ATP stimulation (Figures 5(b) and 5(h)–5(k)). Real-time PCR showed that curcumin substantially decreased mRNA levels of IL-1*β* and IL-18 in pyroptotic microglia (Figures 5(n) and 5(o)). Collectively, our *in vivo* and *in vitro* models revealed that curcumin suppressed stroke-induced NLRP3 inflammasome activation and pyroptosis in microglial cells.

### 3.4. Curcumin Attenuates Microglial Pyroptosis through NLRP3 Inflammasome Inhibition *In Vivo* and *In Vitro*

**Figure 6:**
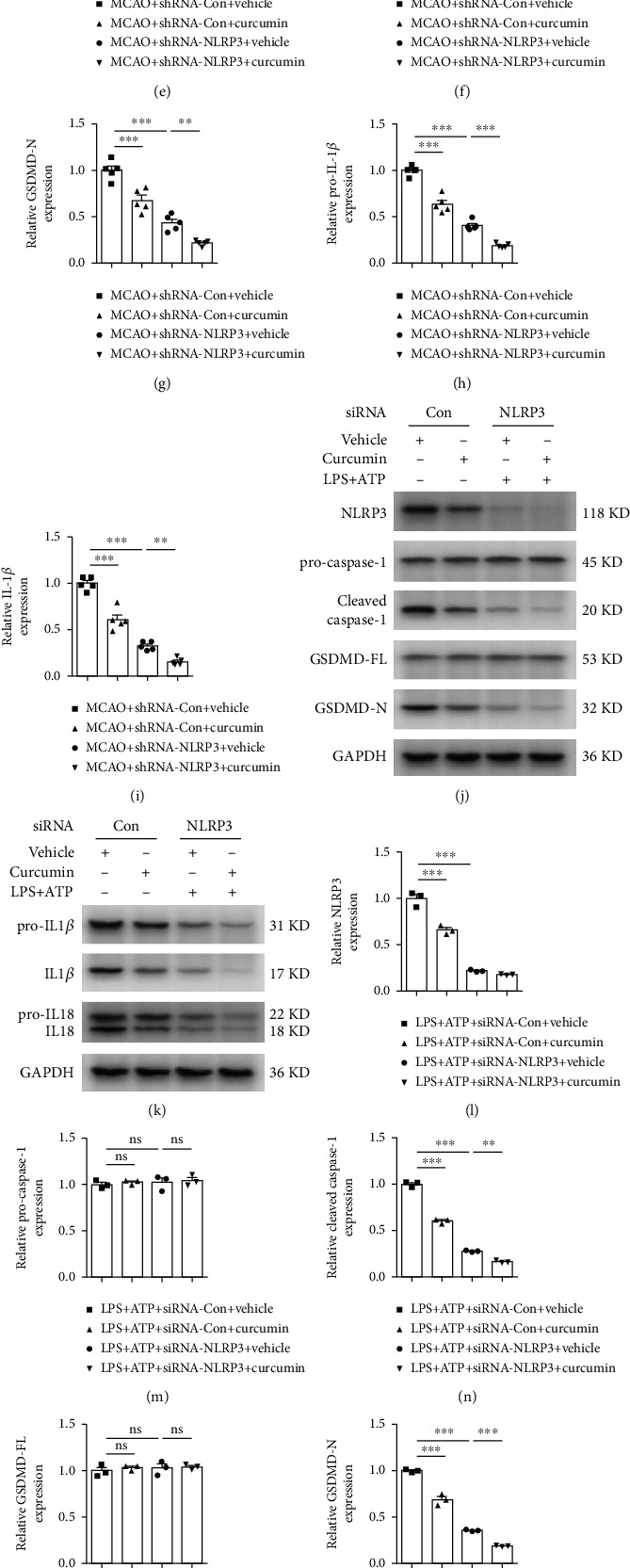
Curcumin and NLRP3 inflammasome inhibition attenuate stroke-induced microglial pyroptosis *in vivo* and *in vitro*. *In vivo*, custom-made AAV vectors carrying shRNA targeting NLRP3 (NLRP3-shRNA) were used to silence the expression of NLRP3 for 14 days, followed by the operation of MCAO. Protein samples were extracted from the ipsilateral peri-infarct areas 21 days after stroke. *In vitro*, NLRP3 was knocked down with siRNA in primary microglia. Representative western blot of pyroptosis-associated proteins *in vivo* (a, b) and *in vitro* (j, k). Quantitative analysis of western blot data for NLRP3 (c, l), pro-caspase-1 (d, m), cleaved caspase-1 (e, n), GSDMD-FL (f, o), GSDMD-N (g, p), pro-IL-1*β* (h, q), IL-1*β* (i, r), pro-IL-18 (s), and IL-18 (t). Values are the mean ± SEM. *In vivo*, *n* = 5 mice per group, 1 band/mouse. *In vitro*, samples were collected from three independent experiments. *n* = 3 per group. ^∗^*p* < 0.05, ^∗∗^*p* < 0.01, and ^∗∗∗^*p* < 0.001, one-way ANOVA followed by Bonferroni post hoc test.

As described earlier, curcumin treatment remarkably reduced NLRP3 inflammasome and consequently ameliorated microglial pyroptosis following stroke (Figures 2–5). To evaluate the relevance of NLRP3 inflammasome inhibition on microglial pyroptosis after stroke, we analyzed the expressions of proteins associated with pyroptosis in the peri-infarct areas following NLRP3 knockdown with AAV-based shRNA (Figures 6(a) and 6(b)). NLRP3 expression was silenced in the brain with NLRP3-shRNA achieving a substantial knockdown of NLRP3 in the peri-infarct areas, as manifested by the decreased NLRP3 protein levels (Figure S1 and Figures 6(a) and 6(c)). We found that either NLRP3 knockdown or curcumin treatment attenuated the levels of pyroptosis-associated proteins, such as cleaved caspase-1, GSDMD-N, pro-IL-1*β*, and IL-1*β* in perilesion regions 21 days after MCAO, compared to the control group (Figures 6(a), 6(b), 6(e), 6(g), and 6(i)). Meanwhile, NLRP3-shRNA treatment markedly enhanced curcumin's effects on the levels of these pyroptosis-related proteins.

Next, we carried out an *in vitro* experiment to further evaluate whether curcumin's antipyroptosis effects are associated with NLRP3 activation. NLRP3 was effectively knocked down by siRNA transduction in resting primary microglia, as evidenced by the western blot result (Figure S2). Similarly, siRNA transduction greatly reduced NLRP3 protein expression in LPS+ATP-stimulated microglia (Figures 6(j) and 6(l)). As illustrated in Figures 6(j), 6(k), and 6(m)–6(t), NLRP3 inhibition with siRNA decreased the levels of pyroptosis-related proteins including GSDMD-N, cleaved caspase-1, pro-IL-1*β*, IL-1*β*, pro-IL-18, and IL-18 in primary microglial cells treated with LPS+ATP. In accordance with our *in vivo* data, curcumin treatment remarkably attenuated microglial pyroptosis in the siRNA-control group stimulated with LPS plus ATP. Interestingly, NLRP3 knockdown enhanced the inhibitory effects of curcumin on microglial pyroptosis.

### 3.5. Curcumin Treatment Reduces White Matter Damage and Improves Functional Outcomes through Inhibition of NLRP3 Inflammasome Activation

**Figure 7:**
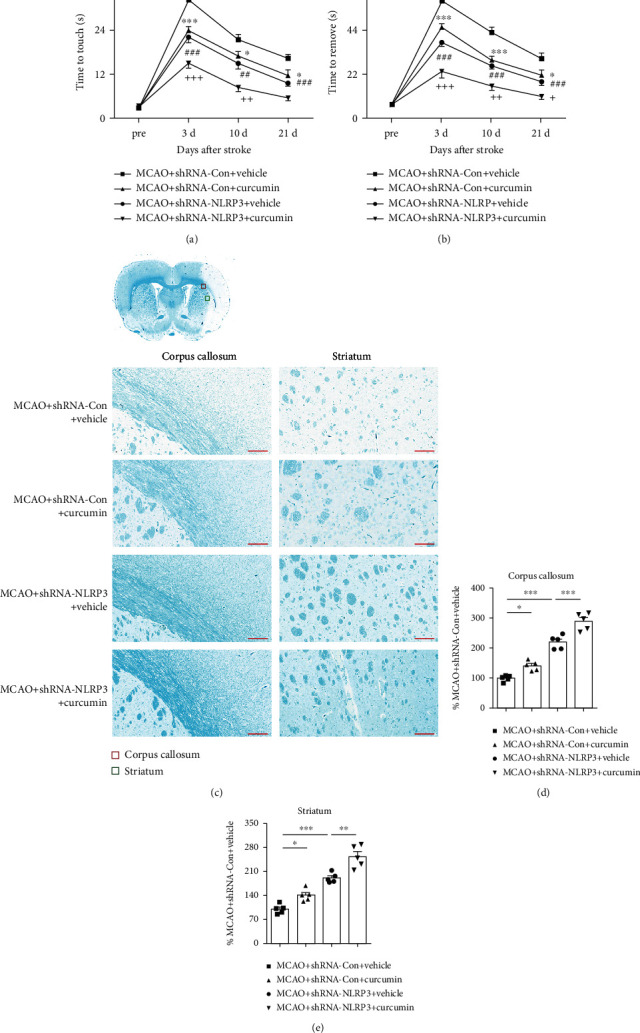
Curcumin treatment and NLRP3 inflammasome inhibition reduce white matter damage and improve functional outcomes following stroke. AAV-based NLRP3 shRNA was used to silence the expression of NLRP3 14 days prior to MCAO operation. (a, b) The adhesive removal was tested at presurgery and 3, 10, and 21 days after MCAO in mice treated with vehicle or curcumin. (a) Time to touch adhesive tapes, (b) time to remove adhesive tapes, *n* = 5 for each group. Data are presented as means ± SEM. ^∗^*p* < 0.05, ^∗∗^*p* < 0.01, and ^∗∗∗^*p* < 0.001, MCAO+shRNA-Con+vehicle *vs*. MCAO+shRNA-Con+curcumin; ^#^*p* < 0.05, ^##^*p* < 0.01, and ^###^*p* < 0.001, MCAO+shRNA-Con+vehicle *vs*. MCAO+shRNA-NLRP3+vehicle; ^+^*p* < 0.05, ^++^*p* < 0.01, and ^+++^*p* < 0.001, MCAO+shRNA-NLRP3+vehicle *vs*. MCAO+shRNA-NLRP3+curcumin. Two-way ANOVA followed by Bonferroni post hoc test. (c) Schematic diagram illustrating the anatomical location of images in the ipsilateral perilesion corpus callosum (red) and striatum (green). Representative LFB staining in the ipsilateral peri-infarct corpus callosum (CC) and striatum areas 21 days after MCAO. Scale bar = 50 *μ*m (CC, STR). (d, e) The integrated density of LFB staining was quantified and normalized to the MCAO+shRNA-Con+vehicle group. *n* = 5 for each group. ^∗^*p* < 0.05, ^∗∗^*p* < 0.01, and ^∗∗∗^*p* < 0.001. One-way ANOVA followed by Bonferroni *post hoc* test.

To measure the significance of NLRP3-mediated pyroptosis in neuropathology and functional recovery after stroke, we next assess white matter damage and sensorimotor functional recovery after MCAO in NLRP3-shRNA knockdown mice. NLRP3-shRNA significantly reduced time to contact and removal of the tapes (Figures 7(a) and 7(b)) and enhanced curcumin's impacts on sensorimotor functional recovery after MCAO. Moreover, the results of LFB staining showed that curcumin-induced protection against white matter damage was markedly enhanced 21 days after stroke in NLRP3-shRNA knockdown mice (Figures 7(c)–7(e)). Finally, NLRP3-shRNA alone greatly attenuated stroke-induced white matter lesions and again enhanced curcumin neuroprotective effects. Our results suggest that curcumin treatment reduces white matter damage and improves functional outcomes, through inhibition of NLRP3 inflammasome activation.

### 3.6. Curcumin Inhibits NF-*κ*B Activation in Microglia after Stroke *In Vivo* and *In Vitro*

**Figure 8:**
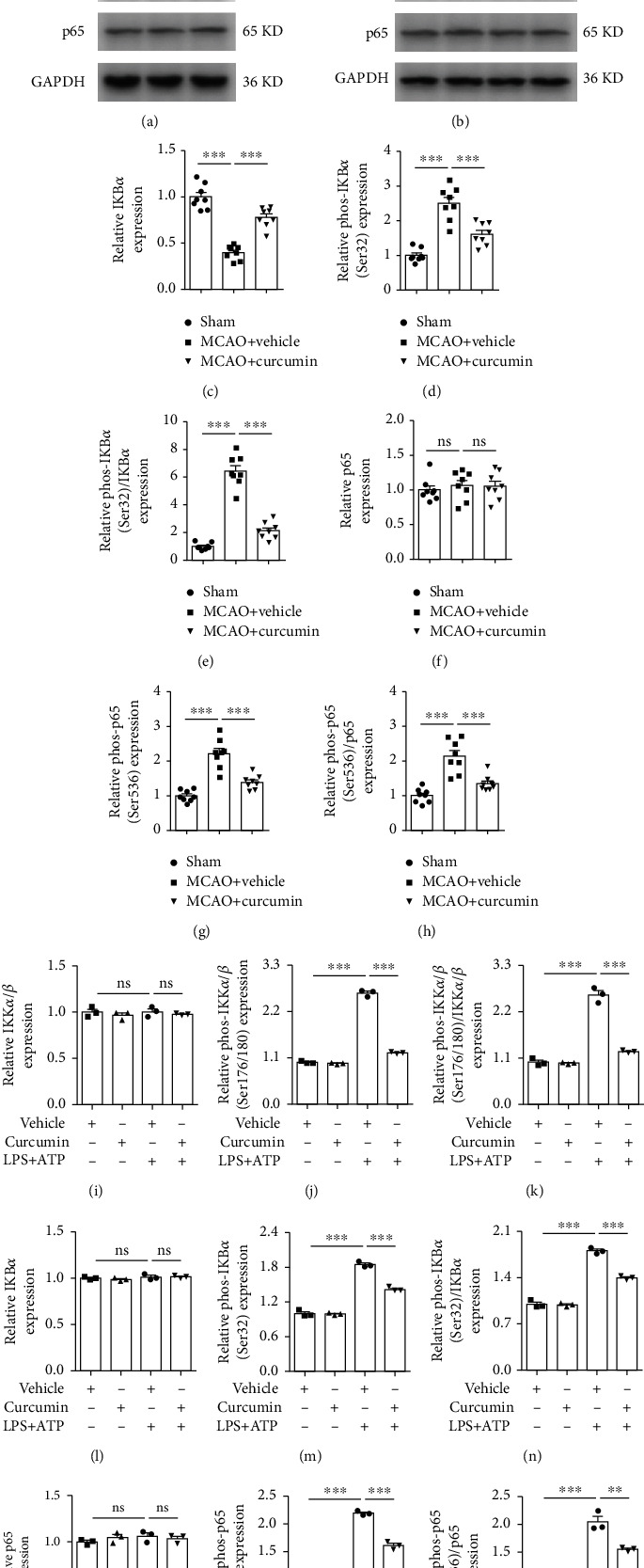
Curcumin treatment inhibits the stroke-induced activation of NF-*κ*B pathway *in vivo* and *in vitro*. *In vivo*, curcumin (150 mg/kg) was intraperitoneally injected into mice after reperfusion and once per day for 7 days after MCAO. Brain samples were collected from the ipsilateral peri-infarct regions 21 days after cerebral ischemia. *In vitro*, primary microglia were induced to pyroptosis with LPS plus ATP after pretreatment with curcumin or vehicle for 24 hours. Representative western blot of NF-*κ*B pathway-related proteins *in vivo* (a) and *in vitro* (b). Quantitative analysis of western blot data for I*κ*B*α* (c, l), phos-I*κ*B*α* (d, m), the relative ratio of phos-I*κ*B*α* vs. total I*κ*B*α* (e, n), p65 (f, o), phos-p65 (g, p), the relative ratio of phos-p65 vs. total p65 (h, q), IKK*α*/*β* (i), phos-IKK*α*/*β* (j), and the relative ratio of phos-IKK*α*/*β* vs. total IKK*α*/*β* (k). Values are the mean ± SEM. *In vivo*, *n* = 8 mice per group, 1 band/mouse. *In vitro*, samples were collected from three independent experiments. *n* = 3 per group. ^∗∗^*p* < 0.01 and ^∗∗∗^*p* < 0.001, one-way ANOVA followed by Bonferroni post hoc test.

Curcumin and NLRP3 knockdown showed a synergistic effect on neuroprotection and sensorimotor functional recovery after MCAO. This is indicative of inflammasome inhibition through modulation of an upstream signaling cascade. The NF-*κ*B pathway triggers a myriad of proinflammatory responses in microglia after brain ischemia, including upregulation of inflammasome components. Therefore, we measured the levels of NF-*κ*B pathway-related proteins, including phosphorylated-I*κ*B*α* (phos-I*κ*B*α*) and phos-p65, in the peri-infarct areas of ischemic mice (Figure 8(a)). MCAO induced NF-*κ*B activation, as manifested by an increase in both the percentage of phos-I*κ*B*α* (Ser32) in I*κ*B*α* (Figures 8(a) and 8(e)) and percentage of phos-p65 (Ser536) in p65 (Figures 8(a) and 8(h)), 21 days after stroke. In contrast, curcumin administration greatly attenuated stroke-induced phosphorylation of I*κ*B*α* and p65 (Figures 8(a), 8(d), and 8(g)). Besides, curcumin significantly reversed the decrease in I*κ*B*α* induced by stroke (Figures 8(a) and 8(c)). *In vitro* experiments were performed to further confirm curcumin's inhibitory effect on NF-*κ*B activation (Figure 8(b)). Consistent with our *in vivo* results, LPS+ATP increased the relative protein expressions of phos-IKK*α*/*β* (Ser176/180) (Figures 8(b) and 8(k)), phos-I*κ*B*α* (Ser32) (Figures 8(b) and 8(n)), and phos-p65 (Ser536) (Figures 8(b) and 8(q)) compared to the vehicle. Contrastingly, curcumin dramatically reduced the impact of LPS+ATP stimulation in the relative protein expressions of phos-IKK*α*/*β* (Ser176/180) (Figures 8(b) and 8(j)), phos-I*κ*B*α* (Ser32) (Figures 8(b) and 8(m)), and phos-p65 (Ser536) (Figures 8(b) and 8(p)) in primary microglia. These findings support that curcumin treatment dramatically suppressed NF-*κ*B activation in microglia following stroke.

## 4. Discussion

In CNS, white matter is composed of myelin sheaths and enwrapped axons [27]. The myelin is wrapped around the internodes of axons, whereby facilitating saltatory conduction of neurological signals. Myelin density, indicated by LFB staining, can denote white matter integrity. In comparison to grey matter, white matter is more susceptible to ischemic insult and more severely damaged, due to limited blood flow and little collateral circulation [28]. Previous preclinical studies mainly concentrated on ischemic neuronal protection. However, protection against white matter damage has often been neglected. Focal cerebral ischemia can induce myelin loss and axonal integrity interruption. Thus, regardless of the protection of neurons in grey matter, loss of myelin and axonal integrity can interfere with neuronal connection [29]. White matter integrity plays an important role in long-term recovery after stroke. Neurological outcomes following stroke were shown to be negatively correlated with white matter lesions in mice [30]. Given the critical association between white matter injury and ischemic stroke, recent studies have focused on therapeutic strategies toward restoring white matter after stroke. A few pharmacological interventions, such as Activin A [30] and cornel iridoid glycoside [31], were shown to provide neuroprotection against ischemic stroke *via* promoting white matter integrity. Up to now, curcumin's neuroprotective effects have focused on stroke-induced grey matter lesions. Here, we investigate the therapeutic effects of curcumin against long-term white matter injury after stroke. Our data revealed a significant improvement in white matter integrity in both CC and striatum areas in mice, accompanied by enhanced sensorimotor function recovery 21 days after stroke. Endogenous oligodendrocyte progenitor cells (OPC) proliferating into myelin-producing oligodendrocytes are located at the CC and the striatum regions after ischemic stroke [32]. Consistent with our research, treatment with curcumin was proved to play a key role in alleviating white matter damage in animal models of hypoxic-ischemic encephalopathy [33] and spinal cord injury [34]. But we further revealed that the neuroprotective effects of curcumin on white matter injuries were associated with microglial pyroptosis.

Microglia-mediated inflammatory responses have a critical impact on the secondary damage and spontaneous repair of white matter after stroke [9]. Oligodendrocytes are major components of white matter. Proinflammatory microglial responses aggravate stroke-induced oligodendrocytes/OPC death and suppress remyelination through proinflammatory cytokine secretion [8, 9]. By contrast, regulating microglial polarization toward an anti-inflammatory phenotype can promote the restoration of impaired white matter *via* the release of neuroprotective factors including TGF-*β* and IL-10 [8]. Several studies reported that the administration of fingolimod [24] or erythropoietin [35] could enhance white matter repair indirectly through the regulation of microglial polarization. Therefore, inhibiting proinflammatory microglial responses is believed to be a potential therapeutic strategy to enhance white matter integrity and improve neurological recovery after stroke. In a previous study, we showed that curcumin indirectly improved functional restoration by modulating microglia/macrophage polarization toward anti-inflammatory phenotype [21]. In the present study, we analyzed the inhibitory effect of curcumin on microglial pyroptosis, a proinflammatory programmed cell death, using an *in vivo* MCAO model and *in vitro* model of primary microglia after LPS+ATP stimulation. Pyroptosis is characterized by caspase-1-mediated cleavage of the N-terminal domain of GSDMD, forming GSDMD pores, thereby enabling the release of proinflammatory IL-1*β* and IL-18. Emerging studies showed that pyroptosis is involved in the pathogenesis of ischemic stroke and pyroptosis suppression ameliorates brain injury [36]. Pyroptosis has been reported in multiple cell types of the CNS such as neurons and microglia [37] following stroke. Our *in vivo* and *in vitro* results indicated that curcumin treatment significantly decreased pyroptosis-related protein levels including GSDMD-N, cleaved caspase-1, IL-1*β*, and IL-18 in microglia. Thus, the beneficial role of curcumin against white matter injury induced by stroke is at least partially attributed to pyroptosis inhibition in microglia. In a rat model of chronic cerebral hypoperfusion combined with diabetes mellitus, curcumin was reported to protect against cognitive impairments partially through pyroptosis inhibition [38].

As discussed earlier, pyroptosis is initiated by inflammasomes and executed by GSDMD. Specifically, NLRP3 inflammasome is known to be one of the most critical mediators of pyroptosis among the various inflammasomes. It plays a crucial role in the pathological process of stroke [39]. NLRP3 inflammasome deficiency or treatment with its selective inhibitor could markedly reduce ischemic brain injury [40]. In particular, stroke was found to evoke microglial pyroptosis-mediated proinflammatory responses *via* the activation of NLRP3 inflammasome [13, 36]. In cerebral ischemia, NLRP3 inflammasome activation evoked the cleavage of caspase-1, thereby cleaving GSDMD, pro-IL-1*β*, and pro-IL-18 to GSDMD-N, mature IL-1*β*, and IL-18, respectively. In the present study, our *in vivo* and *in vitro* data indicated that curcumin greatly inhibited the activation of NLRP3 inflammasome induced by ischemic stroke. Therefore, curcumin's suppression of microglial pyroptosis may be associated with its suppression of NLRP3 inflammasome activation. Here, NLRP3 knockdown with AAV-based shRNA *in vivo* significantly inhibited microglial pyroptosis, decreased white matter damage, and improved sensorimotor functional recovery. Additionally, NLRP3 silencing synergistically enhanced curcumin antipyroptotic and neuroprotective effects in microglial and white matter lesions. These results were confirmed *in vitro* where NLRP3 knockdown significantly enhanced curcumin-mediated pyroptosis inhibition. These findings confirmed that curcumin treatment and inhibition of NLRP3 inflammasome activation ameliorated stroke-induced microglial pyroptosis. In line with our results, curcumin was also found to suppress the levels of proinflammatory mediators including IL-1*β*, IL-18, interferon gamma (IFN-*γ*), and TNF-*α* in a THP-1 macrophage cell line *via* the downregulation of NLRP3 inflammasome activation in a mouse medial meniscus model of osteoarthritis [41]. Collectively, the inhibition of NLRP3 inflammasome activation plays a central role in curcumin's antipyroptosis function in macrophages.

**Figure 9:**
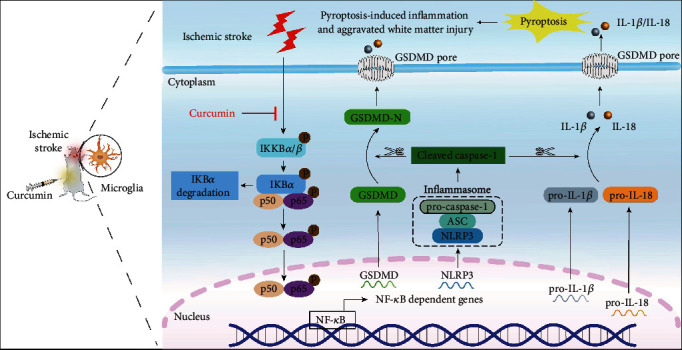
Schematic diagram illustrating the inhibitory effect of curcumin on microglial pyroptosis and proinflammatory responses by NF-*κ*B signaling suppression and NLRP3 inhibition after stroke. Ischemic stroke triggers the activation of NF-*κ*B with the translocation of p50 and p65 into the nucleus in microglia, facilitating the transcription of target genes, such as GSDMD, NLRP3, IL-1*β*, and IL-18. The oligomerization of NLRP3 with the ASC and pro-caspase-1 generates NLRP3 inflammasome, which activates caspase-1. Activated caspase-1 subsequently cleaves the GSDMD, pro-IL-1*β*, and pro-IL-18 into GSDMD-N, IL-1*β*, and IL-18, respectively. GSDMD-N translocates to the plasma membrane and eventually forms membrane pores, leading to the release of IL-1*β* and IL-18. Thus, the microglial pyroptosis-mediated proinflammatory responses aggravate stroke-induced white matter damage. Curcumin dramatically suppresses NF-*κ*B signaling, inhibiting stroke-triggered NLRP3 upregulation and activation and microglial pyroptosis. Consequently, brain inflammation is significantly decreased, reversing white matter damage and function deficits.

Curcumin can attenuate doxorubicin-induced cardiomyocyte pyroptosis *via* NLRP3 inflammasome suppression [42]. Here, we showed that NLRP3 knockdown and curcumin treatment act synergistically to prevent white matter damage, indicative of upstream signal regulation. The multifarious mechanisms including reducing K^+^ efflux, inhibiting Ca^2+^ influx, suppressing NF-*κ*B activation, and preventing the binding of ASC adapter to NLRP3 are involved in curcumin's inhibitory effect on NLRP3 inflammasome activation [43]. In particular, the NF-*κ*B pathway plays a central role in the aggregation of NLRP3 components and the formation of NLRP3 inflammasome activation. NF-*κ*B signaling is the principal mediator of proinflammatory responses, as it is involved in the transcription of proinflammatory factors such as NLRP3, IL-6, TNF-*α*, pro-caspase-1, pro-IL-1*β*, and pro-IL-18. Activated NLRP3 inflammasome further activates caspase-1, which in turn promotes the maturation of proinflammatory cytokines like IL-1*β* and IL-18. In a stroke model *in vitro*, the anti-inflammatory effect of curcumin on brain microvascular endothelial cells was found to be associated with the suppression of the NF-*κ*B signaling pathway [44]. Therefore, we hypothesized that curcumin reduced NLRP3 inflammasome-mediated microglial pyroptosis probably through inhibition of the NF-*κ*B pathway after stroke. In our study, the phosphorylation levels of IKK*α*/*β*, I*κ*B*α*, and p65 were significantly increased after stroke *in vivo* and *in vitro*, compared with the control group, which were remarkably reversed by curcumin treatment. Thus, the inhibition of NLRP3 inflammasome activation by curcumin is mediated through NF-*κ*B signaling suppression in microglia. Our findings are consistent with previous reports showing that the NF-*κ*B signaling pathway was associated with curcumin's repression of NLRP3 inflammasome activation in proinflammatory macrophages [45, 46]. In this report, we did not assess the direct dependency of NF-*κ*B signaling, and it is possible that direct interference with upstream signaling cascades such as TLR could result in similar downstream effects. Despite being widely available, NF-*κ*B knockout mice have pronounced systemic immunological defects making it an impractical model for neurological disease. Nonetheless, recent developments in conditional knockout or knockdown approaches might circumvent this problem. Future studies will directly assess curcumin's dependency on NF-*κ*B signaling for decreased white matter damage following stroke. Altogether, posttreatment of curcumin attenuates stroke-induced white matter damage through suppression of NF-*κ*B signaling and inhibition of NLRP3 inflammasome (Figure 9).

More and more studies have shown that curcumin exerts diverse neuroprotective functions in a direct or indirect manner, including reducing neuronal apoptosis [18], promoting neurogenesis [20], inhibiting white matter injury [34, 47], and suppressing microglia pyroptosis [38]. For a direct neuroprotective role, curcumin significantly ameliorated white matter injury, reduced the loss of preoligodendrocytes, and decreased activated microglia in LPS-treated neonatal rats, which is associated with suppression of inducible nitric oxide synthase (iNOS) and NADPH oxidase (NOX) activation [47]. In the nonhuman primate model of normal aging, long-term curcumin treatment increased grey matter density in cortical ROIs (regions of interests) and improved white matter integrity in limbic, cerebellar, and brain stem regions, providing a neurological basis for the improvements of spatial working memory and motor function in aging nonhuman primates treated with curcumin [48]. Daverey and Agrawal revealed that curcumin treatment protected against spinal cord injury-induced white matter damage *via* NF-*κ*B and Nrf2 crosstalk [34]. Notably, the present study demonstrated that curcumin could mitigate white matter injuries and improved sensorimotor behavioral deficits in ischemic stroke mice. Our *in vivo* and *in vitro* studies confirmed that curcumin showed a direct regulatory effect on microglial pyroptosis. For an indirect neuroprotective function, curcumin indirectly improved cognitive impairments in a rat model of chronic cerebral hypoperfusion combined with diabetes mellitus by suppressing neuroinflammation induced by microglial activation, alleviating apoptosis, and reducing NLRP3-dependent pyroptosis [38], indicating that the inhibition of microglia pyroptosis may be an effective therapeutic strategy for ischemic stroke. Similarly, our studies revealed that curcumin treatment ameliorated white matter injury after ischemic stroke by inhibiting microglia/macrophage pyroptosis through NF-*κ*B suppression and NLRP3 inflammasome inhibition. In agreement with our results, several recent studies demonstrated that curcumin exerted indirect neuroprotective effects through modulating microglia-mediated neuroinflammation [21, 22]. However, the major disadvantage of curcumin is its poor bioavailability, which is manifested by low serum and tissue concentrations regardless of the administration route [49]. The low bioavailability of curcumin is mainly due to its rapid and extensive metabolism in the intestine and liver [50, 51]. Recently, the new drug-delivery system could enhance the bioavailability and pharmacokinetics of curcumin, which would benefit its clinical application. For example, nanoformulated curcumin was highly water-soluble and improved its bioavailability by higher plasma levels, modulated the local inflammatory response, ameliorated glial scar, and protected the white matter against spinal cord injury [52]. Therefore, the new drug-delivery system of curcumin could make this compound an ideal drug candidate for stroke.

## 5. Conclusions

Our data indicated that curcumin treatment can attenuate microglial pyroptosis, enhance white matter integrity, and improve functional outcomes, through NF-*κ*B signaling suppression and subsequent NLRP3 inflammasome inhibition. This study exposes NF-*κ*B signaling and NLRP3 activation as a novel therapeutic target and curcumin as a novel therapeutical agent for stroke.

## Figures and Tables

**Table 1:** The nucleotide sequences of AAV-shRNA-NLRP3 and AAV-shRNA-Con.

Name	Sequences (5′-3′)
AAV-shRNA1-NLRP3	5′-CCGGCCATACCTTCAGTCTTGTCTTCTCGAGAAGACAAGACTGAAGGTATGGTTTTTG-3′
AAV-shRNA2-NLRP3	5′-CCGGCCGGCCTTACTTCAATCTGTTCTCGAGAACAGATTGAAGTAAGGCCGGTTTTTG-3′
AAV-shRNA3-NLRP3	5-CCGGCCACATGACTTTCCAGGAGTTCTCGAGAACTCCTGGAAAGTCATGTGGTTTTTG-3′
AAV-shRNA-Con	5′-CCGGCAACAAGATGAAGAGCACCAACTCGAGTTGGTGCTCTTCATCTTGTTGTTTTTG-3′

**Table 2:** Primer of the target genes for real-time PCR.

Genes		Primers (5′-3′)
NLRP3	Forward	5′-GTGGTGACCCTCTGTGAGGT-3′
	Reverse	5′-TCTTCCTGGAGCGCTTCTAA-3′
Caspase-1	Forward	5′-ACAAGGCACGGGACCTATG-3′
	Reverse	5′-TCCCAGTCAGTCCTGGAAATG-3′
IL-1*β*	Forward	5′-CACCTTTTGACAGTGATGAG-3′
	Reverse	5′-AGCCACAATGAGTGATACTG-3′
IL-18	Forward	5′-CAATGGTTCCTTCATTGAGC-3′
	Reverse	5′-AACAAACAGGAGAAGTTGGT-3′
GAPDH	Forward	5′-AATGGATTTGGACGCATTGGT-3′
	Reverse	5′-TTTGCACTGGTACGTGTTGAT-3′

**Table 3:** The information of primary and secondary antibodies.

Primary antibody	Source	Dilution
GSDMD	ARG41404, Arigo Biolaboratories	1 : 500
IL-1*β*	ab234437, Abcam	1 : 1000
IL-18	#57085, Cell Signaling	1 : 1000
Caspase-1	#89332, Cell Signaling	1 : 1000
NLRP3	ab214185, Abcam	1 : 100
phos-IKK*α*/*β* (Ser176/180)	#2697, Cell Signaling	1 : 1000
IKK*α*/*β*	ab178870, Abcam	1 : 1000
phos-p65 (Ser536)	#3036, Cell Signaling	1 : 1000
p65	#8242, Cell Signaling	1 : 1000
phos-I*κ*B*α* (Ser32)	#2859, Cell Signaling	1 : 1000
I*κ*B*α*	#4812, Cell Signaling	1 : 1000
GAPDH	ab181602, Abcam	1 : 10000

## Data Availability

The data used to support the findings of this study are included within the article and the supplementary information file(s).

## Supplementary Materials

Supplementary Materials Figure S1: knockdown of NLRP3 gene with AAV-based shRNA in mice without cerebral ischemia. (A) Representative western blots of NLRP3 *in vivo*. (B) Quantitative analysis of western blot data of NLRP3. *n* = 3, ^∗∗∗^*p* < 0.001, Student's *t*-test. Figure S2: knockdown of NLRP3 gene with siRNA in resting primary microglia. (A) Representative western blots of NLRP3 *in vitro*. (B) Quantitative analysis of western blot data of NLRP3. *n* = 3, ^∗∗∗^*p* < 0.001, Student's *t*-test.
