# Supplementary material for: Curcumin Ameliorates White Matter Injury after Ischemic Stroke by Inhibiting Microglia/Macrophage Pyroptosis through NF-κB Suppression and NLRP3 Inflammasome Inhibition
Source: Oxid Med Cell Longev. 2021 Sep 30;2021:1552127. doi: 10.1155/2021/1552127 (PMC8497115; doi:10.1155/2021/1552127)
Supplement: Supplementary Materials — Figure S1: knockdown of NLRP3 gene with AAV-based shRNA in mice without cerebral ischemia. (A) Representative western blots of NLRP3 in vivo. (B) Quantitative analysis of western blot data of NLRP3. n = 3, ∗∗∗p < 0.001, Student's t-test. Figure S2: knockdown of NLRP3 gene with siRNA in resting primary microglia. (A) Representative western blots of NLRP3 in vitro. (B) Quantitative analysis of western blot data of NLRP3. n = 3, ∗∗∗p < 0.001, Student's t-test. [file 1552127.f1.doc]

**Supplementary information**


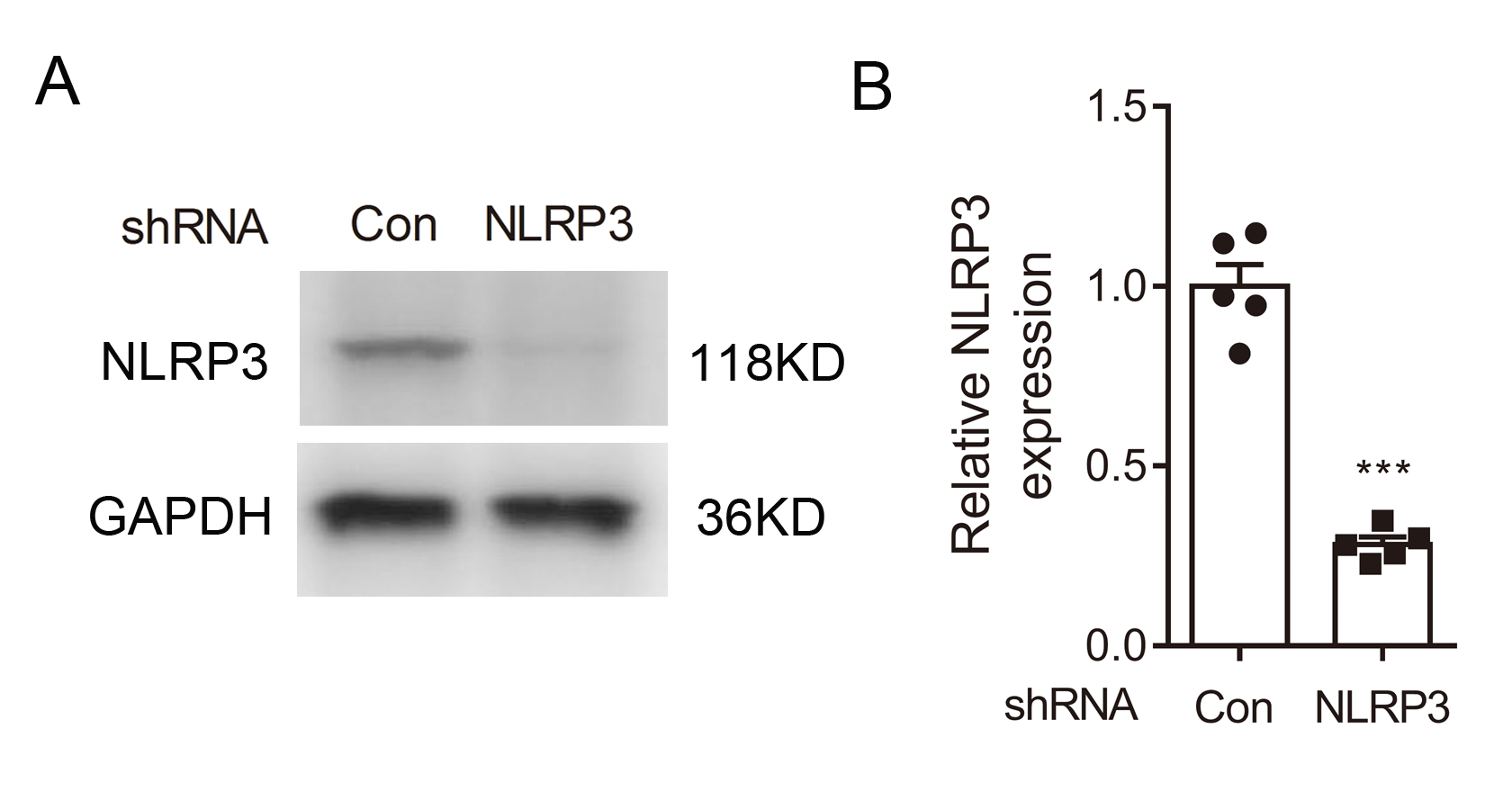


Figure S1. Knock down of NLRP3 gene with AAV-based shRNA in mice without cerebral ischemia. (A) Representative western blots of NLRP3 *in vivo*. (B) Quantitative analysis of western blots data of NLRP3. n = 3, ****p* < 0.001, Student’s *t*-test.


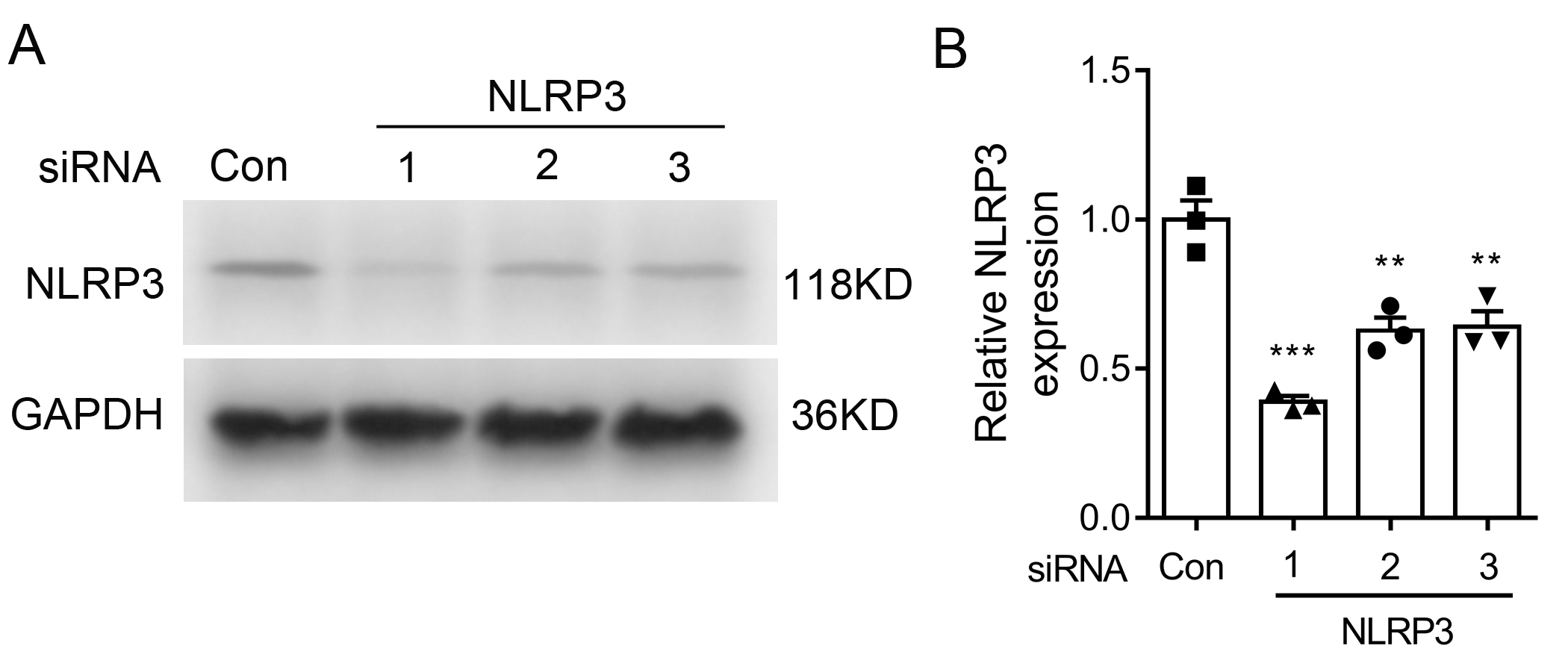


Figure S2. Knock down of NLRP3 gene with siRNA in resting primary microglia. (A) Representative western blots of NLRP3 *in vitro*. (B) Quantitative analysis of western blots data of NLRP3. n = 3, ****p* < 0.001, Student’s *t*-test.
